# Health status of free‐ranging pure and cross‐mixed miniature swine population from Northeast Spain

**DOI:** 10.1002/vms3.665

**Published:** 2021-11-17

**Authors:** Vicente Soler, Encarna Casas, Francesc Closa‐Sebastià, Albert Sanz, Jaume Martorell

**Affiliations:** ^1^ Servei d´Animals Exòtics Fundació Hospital Clínic Veterinari Universitat Autònoma de Barcelona Barcelona Spain; ^2^ Vets and Wildlife Terrasa Spain; ^3^ Servei de Prevenció en Salut Animal Departament d´Agricultura Ramaderia Pesca i Alimentació Generalitat de Catalunya Gran Vía de les Corts Catalanes Barcelona Spain; ^4^ Departament de Medicina i Cirurgía Animals Facultat de Veterinaria Universitat Autònoma de Barcelona Barcelona Spain

**Keywords:** health status, hybrid, miniature pig, serology, Spain

## Abstract

**Background:**

Miniature pigs have gained popularity as companion animals in the recent years in Spain. Due to the fact that their abandonment and crossing breeds with wild boars can cause severe problems, investigation about the health status is needed.

**Objectives:**

The aim of this study was to determine their health status according to the clinical findings during physical examination and the results of antibody serology tests against selected infectious diseases.

**Methods:**

Two‐hundred and eleven miniature pigs (*Sus scrofa*) were included in the study. Their origin, age, sex, housing conditions and diet were recorded.

**Results:**

The housing of the animals ranged from wild animals to ones living in animal sanctuaries. The diet varied from a natural one in the wild to commercial and homemade food. Thirty animals out of two‐hundred and eleven were hybrids between miniature pigs and wild boars according to morphological characteristics. Antibody serology techniques of Influenza A virus, Hepatitis E virus, brucellosis, tuberculosis, African swine fever, Classical swine fever and Aujeszky's disease was performed. The prevalence for Influenza A was 5.30%, for Hepatitis E was 5.35% and the rest tested negative. It is important to control and monitor these zoonotic infections to prevent Public Health problems.

**Conclusions:**

The results obtained from this investigation demonstrated that the animals' health status in this study is optimal and the diseases prevalence is similar or minor when compared to previous studies. This study confirms the hybridization of miniature pig and wild boar in Catalonia.

## Graphical abstract

This paper describes a research project about health status of free‐ranging pure and cross‐mixed miniature swine population from North East Spain based on analysis of diet, environment, clinical findings, physical examination, and antibody serology techniques.

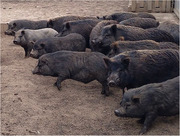

## 1 INTRODUCTION

Miniature pigs (*Sus scrofa domesticus*) are subspecies of the domestic pig. There are 45 different breeds of miniature pigs described worldwide (Amalraj et al., 2018). They have gained popularity as companion animal in the recent years (Sipos et al., 2007).

Miniature pigs’ husbandry depends on the captivity purposes, and environment enrichment is essential to avoid boredom and to prevent destructive behaviour (Amalraj et al., 2018). Mini pigs are omnivorous, but a restricted low energy diet is recommended in pigs kept as pets to prevent obesity. The most appropriate diet for miniature pigs should be approximately 12% protein, 2% fat and 12%–15% fibre (Tynes, 1999). The most common problems observed in pet minipigs are parasitic infections, skin disorders, gastrointestinal, urogenital, respiratory, reproductive tract disorders and locomotor problems (Amalraj et al., 2018; Sipos et al., 2007).

Currently, the abandonment of miniature pigs and crossing breeds with wild boars can cause problems in public health since the animals inhabit in urban or semi‐urban areas where their control and eradication is difficult, thus requiring more strict regulations to control ownership to prevent uncontrolled breeding and infection among wild boars, semi‐wild pigs and farm pigs (Delibes–Mateos & Delibes, 2013; Royal Decree 1392/2012). Notifiable diseases in pigs in Spain include, among others brucellosis, African swine fever (ASF), classical swine fever (CSF) and Aujeszky's disease (AD).

Due to the lack of information on infectious diseases in miniature pigs, the data have been extrapolated from farm pigs (VanderWaal & Deen, 2018) and from previous studies in wild boar (Closa‐Sebastià et al., 2011).

Brucellosis is an important human pathogen causing zoonosis (Pilo et al., 2015). In pigs, brucellosis can provoke abortions, infertility and orchitis (Escobar et al., 2013).

ASF includes per‐acute, acute, sub‐acute and chronic manifestations that can result in skin erythema, pulmonary oedema, splenomegaly, haemorrhagic lymphadenitis, petechial haemorrhages in lungs, urinary bladder and kidneys. Other clinicals signs are delayed growth, emaciation, joint swelling and skin ulcers (Yoo et al., 2020).

CSF produces marked fever, loss of appetite and lethargy. Haemorrhage in the kidneys, digestive tract lymph node, urinary bladder and necrosis in the tonsils and spleen is also observed (Sun et al., 2011).

AD provokes respiratory, reproductive and neurological disorders in pigs and wild boars (Cano‐Terriza et al., 2019).

The rest of the diseases tested in this serological survey were based on its zoonotic potential including swine influenza, hepatitis E and tuberculosis (TB).

Swine influenza A virus (IAV) is antigenically related to human influenza virus (Crisci et al., 2013). Typical clinical signs are characterized by fever of short duration, inappetence, lethargy, coughing, dyspnoea and nasal discharge.

Hepatitis E virus (HEV) is the most common cause of acute viral hepatitis worldwide in humans. The virus is normally apathogenic in pigs but can enhance the pathogenicity of other viruses (Rose et al., 2011).

Tuberculosis: *Mycobacterium complex* is a multi‐host disease that is shared among farm animals, wildlife and sporadically humans causing granulomatous lesions mainly in respiratory and gastrointestinal tract (Pérez de Val et al., 2017).

The aim of this study is to perform antibody serology techniques against Brucellosis, IAV, HEV, TB, ASF, CSF and ADV to evaluate the conditions of origin housing and diet to know the minipig health status to prevent zoonotic infections and to demonstrate the existence of hybrids of miniature pigs and wild boars in a North‐East region of Spain.

## 2 MATERIALS AND METHODS

**FIGURE 1:**
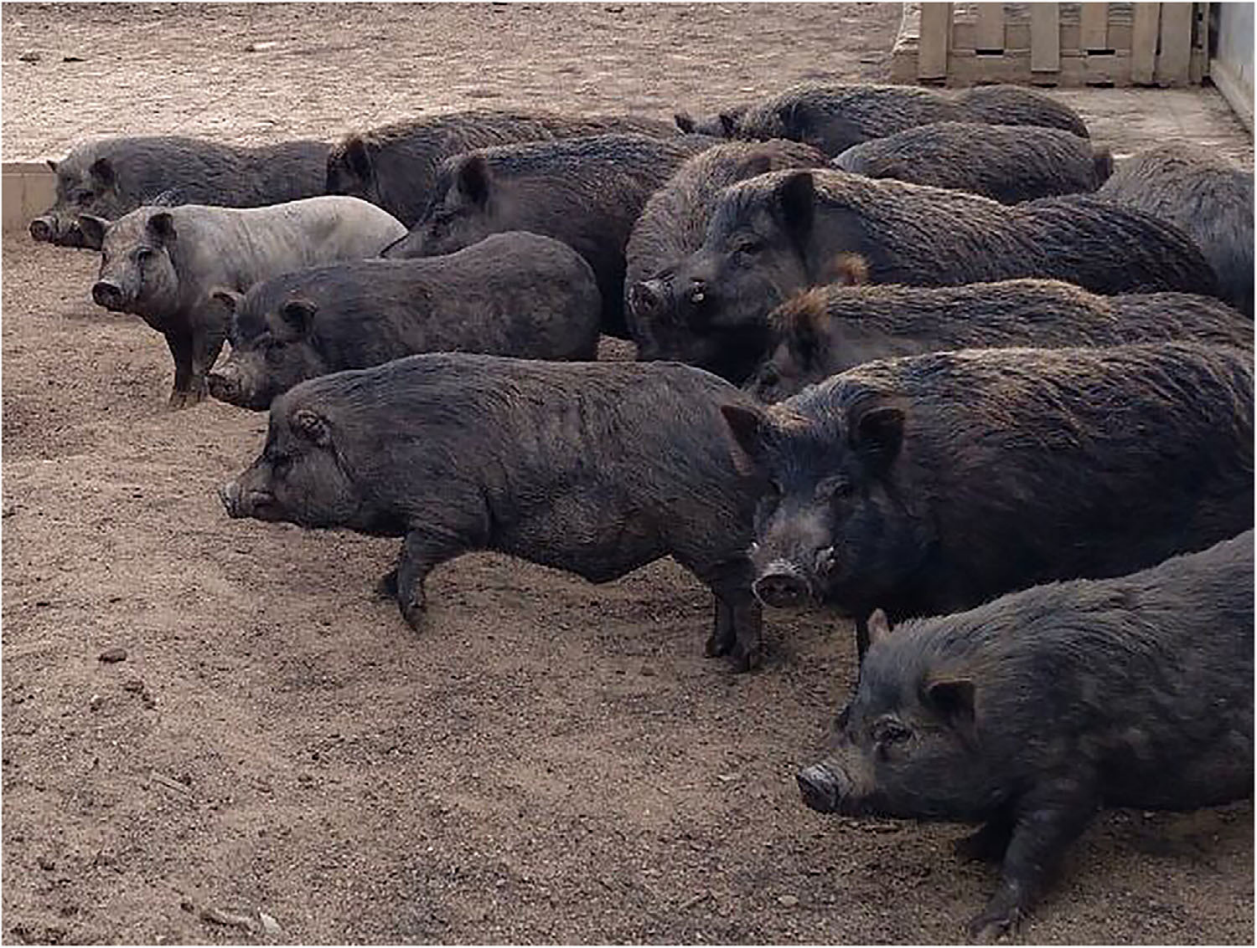
Hybrids showing morphological characteristics of wild boar and miniature pig

**FIGURE 2:**
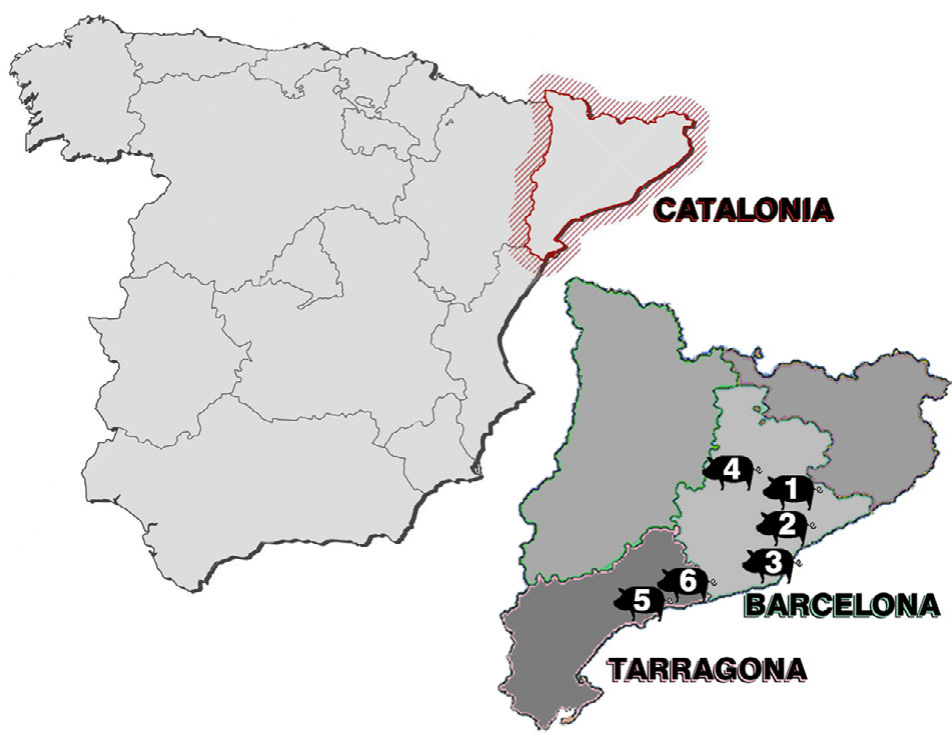
Geographical distribution of the animals sampled in this study in the provinces of Barcelona and Tarragona, Catalonia, Spain. 1: Vallés Oriental. 2: Vallés Occidental. 3: Baix Llobregat. 4: Bages. 5: Tarragonés. 6: Baix Penedés

This study included 211 miniature pigs of different ages and sexes from a North‐East region of Spain (Catalonia) during December 2018 to January 2020. Thirty animals out of two hundred and eleven were hybrids between miniature pig and wild boar according to morphological characteristics (Delibes–Mateos & Delibes, 2013). Hybrids may show characteristics of both animals as a more compact body and longer legs, longer hair or even absence thereof, long snout or also appearance of animals with flat snout (Figure 1). All the animals came from the provinces of Barcelona (Vallés Oriental, Vallés Occidental, Baix Llobregat and Bages) and Tarragona (Tarragonés and Baix Penedés) (Figure 2). The origin of the animals ranged from free‐living in the wild to pigs rescued from different animal sanctuaries. Data collection was divided in sex, age, feeding, housing, region of Catalonia and relevant clinical findings. The animals were divided in three age categories: piglet (less than 3 months), young adults (between 3 and 6 months) and adults (more than 6 months). The animals’ diets were classified according to the information collected from the animal sanctuaries and the veterinarians participating in the study as commercial food (pellet and seed mixture), natural diet from the environment (fresh fruit, vegetables and tubers) and homemade food (food scraps and human waste products). According to the housing conditions, pigs were classified as miniature pigs living in natural lands or forests (free‐living in the wild), outdoor concrete soil (urban, semi‐urban areas and agricultural land) and outdoor enclosure (animal sanctuaries).

All wild animals and sanctuary animals in this study were caught using containment techniques such as nets or fencing approved by an ethics committee. This study was performed under the supervision and following the ethics guidelines of the *‘*Servei de Prevenció en Salut Animal’, Departament d´Agricultura, Ramaderia, Pesca i Alimentació. Generalitat de Catalunya. An informed owner consent was obtained from the director of each sanctuary.

All the individuals were sedated using a combination of tiletamine‐zolazepam (6 mg/kg, Zoletil, Virbac Salud Animal, Espluges de Llobregat, Spain) and xylazine (3 mg/kg, Xilagesic 20%, Calier Laboratories, Les Franqueses del Vallés, Spain) administered intramuscularly. Physical examination included determination of body condition, hydration status, abdominal palpation, palpation of the lymph nodes and cardiac and pulmonary auscultation. Fifteen millilitres of whole blood were obtained using an 18 Ga 1 ^½ ´´^disposable needles (Sterican, Bbraun, Rubí and Spain) and 20 ml syringes (Omnifix, Bbraun, Rubí and Spain) from the caudal cava vein of each pig. Serum samples were submitted to external laboratories for antibody serology detection. Sera were stored at −20°C until analyses.

TB tests were performed by Centre de Reserca de Sanitat Animal, Bellaterra, Spain (CReSA), and the rest of serologies were sent to Laboratori de Sanitat Animal de Catalunya, Lleida, Spain (LaSAC). Serological details are presented in Table 1.

**TABLE 1:** Serological information according to disease, laboratorial technique and trademark

Disease	Laboratorial technique	Trademark
Tuberculosis	ELISA	Nunc‐Maxisorp; Thermo Fisher Scientific, Roslilde, Denmark
Influenza A	Indirect ELISA	INgezim Influenza Porcina Ingenasa Madrid, Spain
	Blocking ELISA	Idexx Influenza A Idexx, Westbrook, Maine, USA
Hepatitis E	Indirect ELISA	PrioCheck HEV Ab porcine Prionics AG, Neuried, Germany
Brucellosis	Bengal Rose	
	Complement fixation	
African swine fever	Blocking ELISA	INgezim PPA Compac Ingenasa, Madrid, Spain
Classical swine fever	Blocking ELISA	Idexx CSFV Ab Idexx, Westbrook, Maine, USA
Aujeszky	Blocking ELISA	Civtest Suis ADV gE Hipra, Gerona, Spain

The prevalence of the diseases was estimated from the ratio of positive samples to the total number of samples analyzed, with confidence intervals of 95%.

## 3 RESULTS

The weight of the animals in the study ranged from 4 to 80 kg (26 ± 19.64 kg). Physical examination, including determination of body condition, hydration status, abdominal palpation, palpation of the lymph nodes and cardiac and pulmonary auscultation, was unremarkable, but different relevant clinical findings were recorded. Two animals presented nail overgrowth, seven animals had scabs (3.31%), 16 females were pregnant and three males were cryptorchid. Geographical distribution, sex, diet, age and clinically relevant findings are presented in Tables 2 and 3.

**TABLE 2:** Animal distribution according to origin, sex, housing, diet and relevant clinical findings of the animals sampled in the study

		Origin		Sex				Housing			Diet			Relevant clinical findings	
		BCN	TAR	♂	♀	♂N	♀S	A	B	C	COM	NAT	HOM	Yes	No
Miniature pigs	Piglet	5	9	13	1				9	5	9		5		
	Young adult	39	32	33	20	12	6	37	19	15	17	31	23	21	
	Adult	57	39	46	37	3	10	52	44		23	50	23	7	
Hybrids	Piglet														
	Young adult	14	1	6	9			6	2	7	2	3	10		
	Adult	6	9	11	4			7	8		7	8			
Total		121	90	109	71	15	16	102	82	27	58	92	61	28	

*Note*: A, forest; B, outdoor cement; C, outdoor enclosure.

Abbreviations: BCN, Barcelona; COM, commercial; HOM, homemade; NAT, natural; TAR, Tarragona.

**TABLE 3:** Animal distribution according to municipality and province of the animals sampled in the study

	Barcelona				Tarragona	
	VO	VOC	BL	BA	TAR	BP
Miniature pigs	33	5	54	9	40	40
Hybrids	2	11	7			10
Total	35	16	61	9	40	50

Abbreviations: BA, Bages; BP, Baix Penedés; BL, Baix Llobregat; TAR, Tarragonés; VO, Vallés Oriental; VOC, Vallés Occidental.

Two hundred and eleven animals were included in the present study. Thirty animals out of two hundred and eleven were hybrids between miniature pig and wild boar. Serological results are presented in Table 4.

**TABLE 4:** Results of serologies, affected animals and number of tests performed in the present study

Disease	Affected animals (%)	Number of tests performed
Tuberculosis	0%	136
Influenza A	5.30% (four out of six in animal sanctuaries)	113
Hepatitis E	5.35% (five out of six in animal sanctuaries)	112
Brucellosis	7.14% Bengal Rose	210
	0% complement fixation	15
African swine fever	0%	211
Classical swine fever	0%	211
Aujeszky	0%	209

## 4 DISCUSSION

This study evidences the presence of pet pigs stablished in the wild due to deliberate or accidental abandonment, resulting in establishments in new colonies (Delibes–Mateos & Delibes, 2013). The presence of pregnant females, piglets and hybrids in the study demonstrated the hybridization between miniature pigs and wild boars in Catalonia, since animals of different age, sex and species were found coexisting but not being castrated.

Despite the different kind of diet between free‐ranging animals and animals living in sanctuaries, no signs of obesity or thinness were observed, considering all the animals in a proper body condition.

A low percentage of skin lesions as scabs, scars and papules have been found (seven out of 211 animals), despite the fact that pigs can be aggressive when sharing small enclosures (Amalraj et al., 2018). In the current study, only two animals out of 211 animals (0.94%) presented overgrown nails. In miniature pigs, lameness is often associated with a poor husbandry and soft surfaces that do not allow an adequate hoof wear (Tynes, 1999). This issue can be solved with the use of more abrasive surfaces and nail trimming (Amalraj et al., 2018; Tynes, 1999). All the animals of the study with nail overgrowth were outdoor concrete soil and outdoor enclosure. Tusk overgrowing is another problem that can occur in miniature pigs, as this grows throughout the life of the animal (Tynes, 1999). This item has not been shown in the study.

Influenza A virus (IV) can infect a wide range of species, including human beings, and remains one of the major threats to human health. Pigs are one of the hosts for the virus and play an important role in its epidemiology (Crisci et al., 2013). Pigs are susceptible to IV from avian and human viruses and can act as reservoirs of the disease for humans, stablishing themselves as zoonoses (Crisci et al., 2013). In fact, the origin of several IV pandemics such as the ones that took place in 1957, 1968 and 2009 were traced down to pigs (Garten et al., 2009 ). In a previous study of farm pigs from Spain, the seroprevalence of swine influenza ranged from 23.4% to 87.3%. In the case of the region of Catalonia, the virus reached 79.7% (Simon‐Grifé et al., 2011) while in wild boars it was about 6.4% (Closa‐Sebastià et al., 2011). From the results of this study, only 5.3% of the animals tested positive for influenza virus, four animals out of six positives belonged to animal sanctuary, increasing the risk of transmission with caretakers of the centre. Two animals out of the six positive ones, were free‐living animals, enhancing the dissemination of this disease in wildlife populations as seen in previous reports (Closa‐Sebastià et al., 2011; Simon‐Grifé et al., 2011). This result is lower than in previous reports done in farm pigs and wild boars. The best way to control the disease is surveillance plans.

HEV is an infectious disease present in wild boars and domestic pigs (Rose et al., 2011). This disease is considered an emerging zoonosis in many developing countries. As opposed, in industrialized countries, it occurs sporadically (Pavio et al., 2010). This virus is known to be responsible for 20 million of new infections, 3.3 million of acute hepatitis E and 44,000 deaths from acute liver failure in human beings every year (Sooryanarain & Meng, 2020). It is suggested that zoonosis can happen through meat consumption and/or contact with infected wildlife (Rivero‐Juarez et al., 2018) or direct contact with infected pigs (Sooryanarain & Meng, 2020). Pigs are a natural reservoir for the HEV (Salines et al., 2017). It is reported that mingling practices, nursery procedures and poor hygiene are risk factors that ease the spread of the disease (Salines et al., 2017). Some experimental studies showed that HEV strains could be transmitted from European wild boars to domestic pigs (Jori et al., 2016 ), but further investigation is needed. A previous report in Europe showed that the prevalence of HEV in wild boars fluctuated between 2% and 68% (Rivero‐Juarez et al., 2018). In the case of Spain, the prevalence varied from 19.6% (de Deus et al., 2008) to 23.2% (Rivero‐Juarez et al., 2018). The present study found a prevalence of Hepatitis E of 5.35%, where five animals out of the six positives ones were from sanctuary, increasing the risk of zoonosis among caretakers, because this disease is asymptomatic in pigs (Rivero‐Juarez et al., 2018). One animal out of six positive animals was in the free‐living group, enhancing the risk of spreading the disease to others, because it was living in wildlife (Sooryanarain & Meng, 2020; Rivero‐Juarez et al., 2018). The best ways to control this disease are wildlife, farm livestock surveillance plans and strict veterinary and sanitary control (Jiménez De Oya et al., 2011).

Brucellosis is a zoonotic infection caused by *Brucella suis*. This pathogen affects domestic and feral pigs (Pilo et al., 2015). Porcine brucellosis is a re‐emerging disease in South‐East Asia and South America and a potential arising infection in some countries since *B. suis* is a common finding in wild boar population and its dissemination from wild boar to outdoor‐reared pigs is reported (Pilo et al., 2015). In case of humans, this disease is associated with direct contact with infected animals, genital secretions and poor hygiene measures (Escobar et al., 2013). The most effective measures to control the disease are complete separation between wild boars and domestic pig population and surveillance of brucellosis in wildlife (Pilo et al., 2015). In the study, two different laboratory techniques were used for the diagnosis of brucellosis according to the World Organization for Animal Health (OIE). The first technique was the Bengal Rose, which allows an initial screening on the field (Farro et al., 2002), 15 animals out of 210 tested positive. Positive samples were then tested with complement fixation test to confirm the presence of *Brucella spp*. Complement fixation technique discriminates non‐specific agglutinins better (Farro et al., 2002). This fact could explain the difference obtained from both tests. All 15 positive samples for Bengal Rose were negative to complement fixation and were therefore sorted out as negative.

TB is an infectious disease caused by a range of *Mycobacterium complex* species. It is a multi‐host disease which can infect farm animals, wildlife and sporadically humans (Pérez de Val et al., 2017). The Eurasian wild boar has been documented as a maintenance host in the Mediterranean countries (Pérez de Val et al., 2017). In 2004, an outbreak of TB in wild boars was detected in Spain. The outbreak was originated in territories where cows and wild boars shared water and food, which resulted in close contact between these two populations and facilitated the spreading of the disease (Hermoso de Mendoza et al., 2006). The prevalence of TB reached 70% in adult wild boar (Mentaberre et al., 2014), another study in wildlife of the Doñana National Park showed a prevalence of 76.7% in wild boar (Barroso et al., 2020). In addition, a previous study in Catalonia revealed a TB prevalence of 7.6% in wild boar (Ciaravino et al., 2020); however, all the samples tested in this study were negative for *Mycobacterium*, being a different result compared to other parts of Spain.

ASF is a high mortality viral disease. The natural hosts are wild suids and arthropods, being asymptomatic in the natural swine reservoir hosts. However, in the domestic pig, the infection is lethal (Galindo & Alonso, 2017). Although the disease was eradicated in Europe in the 1990s with the exception of Sardinia (Italy), different outbreaks in 2007 and 2014 were observed in eastern countries (Galindo & Alonso, 2017). However, Spain is free of this disease. Efficient measures are quarantine and biosecurity, animal movement restrictions and slaughtering affected and exposed animals (Galindo & Alonso, 2017). In previous studies of wild boars in Poland, ASF virus was detected, and this fact may contribute to the spread of this disease (Blome et al., 2012). The European Commission has recommended procedures for the control of ASF in domestic pig and wild boar populations. In the present study, all the samples tested were negative for ASF, which correlates with the results shown in a previous report in wild boars in Catalonia (Closa‐Sebastià et al., 2011).

CSF is one of the most important viral diseases of domestic pigs and wild boars due to its impact on animal health and pig industry worldwide (Blome et al., 2017). Control measures include stamping out of infected herds, delimitation of restriction zones and movement restrictions (Ganges et al., 2020). Another measure described to maintain the disease under control was the vaccination of wild boars because it is known that wild boars are an important reservoir of the virus (Sophie Rossi et al., 2015). A previous study in Bama miniature pigs showed that this breed was highly susceptible to a highly virulent CSF virus strain (Sun et al., 2011). According to the World Organization for Animal Health (2021), CSF has been currently eradicated from Spain. In this study, all the samples taken for CSF tested negative, which was the expected result.

AD is a domestic swine disease with significant economic relevance that causes large losses in the swine industry due to declining production and trade restrictions (Boadella et al., 2012; Meier et al., 2015; Vicente‐Rubiano et al., 2014). Due to eradication programmes, AD does not occur in domestic swine (Vicente‐Rubiano et al., 2014). The natural hosts of this disease are Suidae. It is widely recognized that wild boars can act as a reservoir of AD (Boadella et al., 2012), along with wild boar transmission of the virus to the domestic pig (Vicente‐Rubiano et al., 2014). As a consequence, disease surveillance in wild boar populations is essential (Meier et al., 2015). The highest seroprevalence of AD is in the Mediterranean countries, including Spain. Catalonia is an autonomous community of Spain where wild boar densities are high but the seroprevalence of this disease is low (Closa‐Sebastià et al., 2011). There are contradictions at the bibliographic level as to whether wild boar is a risk factor for AD seroprevalence or not. In a previous study in wild boars, the prevalence of ADV virus was 0.8% and suggested that this pathogen may not circulate in Catalonia (Closa‐Sebastià et al., 2011). This fact can be corroborated with the results of this study since all samples tested were negative for ADV.

The first limitation of this study was that not all animals underwent the same serological tests. It sometimes was due to a lack of sample and other times because they were later incorporated into the study. The second limitation of this study was not to perform genetic markers in the case of hybrids, based only on morphological characteristics. In future studies, these genetic markers will be carried out.

In conclusion, the health status of the miniature pig populations in the study is optimal, both in free‐living wild animals and animals living in sanctuaries, resulting from the strict surveillance plans settled in Spain and Europe focused on prevention, control and monitoring of infectious diseases in farm pigs, wildlife and animal sanctuaries.

## CONFLICT OF INTEREST

The authors declare no conflict of interest.

## AUTHOR CONTRIBUTIONS

*Data collection, data analysis and drafting the manuscript*: Vicente Soler. *Data collection*: Encarna Casas and Francesc Closa‐Sebastià. *Data analysis*: Albert Sanz. *Data collection, data analysis, drafting and revising the manuscript*: Jaume Martorell.

## ETHICS STATEMENT

This study was performed under the supervision and following the ethics guidelines, of the *‘*Servei de Prevenció en Salut Animal’, Departament d´Agricultura, Ramaderia, Pesca i Alimentació. Generalitat de Catalunya. An informed owner consent was obtained from the director of each Sanctuary.

### PEER REVIEW

The peer review history for this article is available at https://publons.com/publon/10.1002/vms3.665

## Data Availability

The authors confirm that the data supporting the findings of this study are available within the article.
